# Has the National Fall in Smoking Rates in Ireland Been Replicated in Cancer Patients? A 5-Year Report

**DOI:** 10.3390/ijerph19042348

**Published:** 2022-02-18

**Authors:** Patricia Fitzpatrick, Nancy Bhardwaj, Ailsa Lyons, Kirsten Doherty, Kate Frazer, Amanda McCann, Vikram Niranjan, Shiraz Syed, Patricia Fox

**Affiliations:** 1 School of Public Health, Physiotherapy & Sports Science, University College Dublin, D04 V1W8 Dublin, Ireland; patricia.fitzpatrick@ucd.ie (P.F.); vikram.niranjan@ucd.ie (V.N.); 2 Department of Preventive Medicine & Health Promotion, St. Vincent’s University Hospital, D04 T6F4 Dublin, Ireland; ailsa.lyons@svuh.ie (A.L.); k.doherty@svuh.ie (K.D.); shirazsyed@svhg.ie (S.S.); 3 School of Nursing, Midwifery and Health Systems, University College Dublin, D04 V1W8 Dublin, Ireland; kathleen.frazer@ucd.ie (K.F.); patricia.fox@ucd.ie (P.F.); 4 Conway Institute of Biomolecular and Biomedical Research, University College Dublin, D04 V1W8 Dublin, Ireland; amanda.mccann@ucd.ie; 5 School of Medicine, University College Dublin, D04 V1W8 Dublin, Ireland

**Keywords:** smoking, prevalence, cancer, patients, Ireland

## Abstract

Smoking among cancer patients leads to poorer outcomes, yet many patients continue smoking. As part of a feasibility study of smoking cessation for cancer patients in Ireland, smoking rates were reviewed. Hospital Inpatient Enquiry (HIPE) data on the smoking status of discharges with a cancer diagnosis (overall, breast, lung, cervical and head and neck cancer) were used (2014–2018). During 2014–2017, current smoking increased for overall (10.5–11.7%) and lung cancer (24.7–27.2%), then decreased to 11.4% and 24.1%, respectively, in 2018. Current smoking increased for cervical during 2014–2018 (11–19.8%) and initially (2014–2016) for head and neck (3–12.7%) cancer, decreasing to 7.6% in 2018; breast cancer was stable at 6 ± 0.6%. These rates are lower than the Irish (23–20%) and European (29% (average)) general population. During 2014–2017, past smoking increased among overall (15.2–21%) and specific cancers, which was lower than the Irish general population (23–28%). Current smoking was highest among 50–59-year-olds (14–16%), which contrasts with the Irish general population (24–35 years at 32–28%). HIPE data are subject to potential duplicate episodes of care and under-documentation of smoking. However, trend analysis is useful, as these limitations should be stable. Rates remain high; therefore, robust documentation and smoking cessation referrals for cancer patients are important.

## 1. Introduction

Tobacco smoking has been causally linked as an independent risk factor in the pathogenesis and development of a wide variety of cancers [1]. However, the implications of continued smoking after a cancer diagnosis are equally deleterious and the benefits of smoking cessation after a cancer diagnosis are often undervalued [2]. Tobacco smoking is associated with an increased risk of recurrence [3,4,5], the development of second primary cancers [3,6,7,8], poorer treatment outcomes (including treatment-related complications and toxicities) [3,4,9], lower survival rates and decreased quality of life [3,10], and pulmonary, cardiovascular and wound complications leading to increased length of hospital stay [11,12]. Mortality among lung cancer patients is higher with increased smoking and home/work second-hand smoke exposure [13]. There is a growing body of evidence that smoking cessation improves outcomes following the diagnosis of several different cancers [8,14,15]. Some of these smoking-related complications are seen to decrease by 19% with each week of cessation [11]. Notwithstanding the adverse outcomes, a significant number of people who have been diagnosed with cancer continue to smoke [10,16]. Some of the important issues to be addressed to promote smoking cessation among cancer patients include: (i) a lack of awareness of smoking harms, (ii) lack of tailored interventions to promote smoking cessation among cancer patients [2], (iii) limited opportunities for conversation around smoking cessation [17] and (iv) suboptimal use of smoking cessation interventions by cancer patients [17].

Even though approximately 69% of tobacco smokers express a desire to quit and 52% have attempted to quit in the previous year [18], only 4–7% are able to quit without the use of smoking cessation supports [19]. Although patients with head and neck (H and N) and lung cancers (LC) have shown a higher motivation to quit with quit rates as high as 70%, they also have high relapse rates of up to 50% within 6 months [19,20].

Evidence-based smoking cessation supports with consideration to the special needs of cancer patients are therefore needed. As part of a feasibility study, with the objective of assessing the development of a hospital-based, high-intensity, peri-treatment behavioural smoking cessation programme as part of routine care for cancer patients throughout Ireland, a review of smoking rates among cancer patients in Ireland was undertaken.

## 2. Materials and Methods

Rates of current and past smoking during a five-year study period among cancer patients in Ireland were evaluated using Hospital Inpatient Enquiry (HIPE) data. HIPE is a database which collects administrative, demographic and clinical data on discharges from, and deaths in, Irish acute public hospitals [21]. HIPE data were requested for the five most recent years available (2014–2018). Data were received as summary tables representing number of discharges (17 years and older) with a principal diagnosis of cancer, for all cancer types combined (overall) and individual cancers (including breast cancer (BC), lung cancer, cervical cancer (CC) and H and N cancer), sorted by age, gender and smoking status (current and past smoking). Cells with five or fewer discharges were suppressed and not disclosed for reasons of confidentiality. HIPE discharges are coded using the *International Statistical Classification of Diseases and Related Health Problems, 10th Revision, Australian Modification* (ICD-10-AM); *Australian Classification of Health Interventions* (ACHI); and *Australian Coding Standards* (ACS), *8th Edition* [21].

Smoking is collected using ICD-10-AM diagnosis codes which are coded as an additional diagnosis. In the HIPE data, ‘current smoking’ was documented using code Z72.0 ‘Tobacco use—current’, which indicated if the patient has smoked tobacco (any amount) within the last month or there was documentation of ‘hazardous use’ (a pattern of substance use that increases the risk of harmful consequences for the user) of tobacco. Current smoking in our dataset also included code F17.1 ‘harmful use’ of tobacco (the clinician has clearly documented a relationship between a particular condition(s) and smoking (even if the patient has ceased smoking)), and code F17.2 ‘tobacco dependence syndrome’. ‘Past smoking’ (code Z86.43) was documented if the patient had smoked tobacco (any amount) in the past but excluding the last month [22].

Prevalence over five years (2014–2018) was calculated for overall, age-specific and gender-specific smoking rates for patients with all cancer types combined, BC (female), LC, CC and H and N cancer. The relative change in the rate of current smoking from 2014 to 2018 among males and females (overall cancers) was calculated (2014 rate − 2018 rate/2014 rate) × (100). The smoking rates among cancer patients were compared to the general population in Ireland (using ‘Healthy Ireland’ survey reports, a national health and lifestyle survey in people aged 15 and over in Ireland) [23] and in Europe (European Tobacco Use: Trends Report 2019) [24] and with the global trends of smoking among cancer patients. The study hypothesis was that smoking rates in cancer patients in Ireland do not follow the same trend as seen in the general population.

## 3. Results

The total number of discharges with a primary diagnosis of malignancy for all cancer types combined and for individual cancers reported by HIPE during 2014–2018 are presented in Table 1. From 2014 to 2018, discharges with a BC diagnosis were highest (range 8.8–9.0%) compared to other cancers (LC (range 3.1–3.6%), CC (range 1.6–1.4%) and H and N (1%)).

There was an increase in current smoking from 10.5 to 11.7% among cancer patients (overall) from 2014 to 2017 (Table 2). There was a similar rise in the rate of past smokers among cancer patients (overall) from 15.2 to 21% during the same period (Table 3). There was a minor decrease of 0.3% in both current and past smoking in 2018 (Table 2 and Table 3).

Rates of current and past smoking for all cancers combined and individual cancers overall, gender-specific and by age group are shown in Table 2 and Table 3. Current and past smoking rates from 2014 to 2018 were consistently higher among males than females among all types of cancers, with the exception of current and past smoking in H and N cancer in 2017 and 2015/2018, respectively. Current smoking rates among overall cancer patients were higher in males than females (2014–2018). However, the relative increase in the current smoking rate was higher among females (9.1%) than males (7.6%) during 2014–2018. Current smoking rates were consistently higher among the 50–59 years old age group (range 14–15.9%), whereas past smoking rates were consistently higher among the 70 years and older age group (range 17.5–24.5%) for all types of cancers (overall) during 2014–2018. Among all cancer types, LC patients had the highest rate of current (range 24.7–24%) and past smoking (range 30.3–38%), whereas BC patients had the lowest rates of current (range 5.7–6.6%) and past smoking (7.2–12.8%) during this period.

## 4. Discussion

The proportion of survivors has increased among cancer patients and, with increased survival, more attention is now directed to survivorship [25]. The present analysis aimed to estimate the prevalence of smoking among cancer patients admitted to Irish public hospitals during 2014–2018. Current smoking rates among cancer patients overall increased during 2014–2017, followed by a small reduction of 0.3% in 2018. Current smoking was consistently highest among 50–59-year-old cancer patients and among males during 2014–2018. Current smoking rates were highest among LC patients and lowest among BC patients. The rates found are relatively low compared to the general populations (overall and gender-specific) of Europe and Ireland. Almost 29% of the population smokes tobacco in Europe [24], with the prevalence of current smoking ranging from 13–46% across countries during 2016 [24]. A minor fall of 0.3% was observed among cancer patients in 2018; in contrast, smoking rates in the Irish general population fell by 6% (from 23 to 17%) between 2015 and 2019 [26]. In the present analysis, current smoking was higher in males than females in overall cancer patients during 2014–2018 and in most years in LC patients. The upward trend was seen similarly in both genders. Similar gender differences are seen in the overall European and the Irish general populations [23,24]. In this study, current smoking was highest in the 50–59-year-old age group (range 14–16%), older than the Irish general population highest prevalence age group of 25–34 years [23]. These results point to our study hypothesis that smoking trends in cancer patients in Ireland do not follow that seen in the general population.

While smoking among the general population is widely evaluated in developed countries, there are few studies evaluating smoking trends among cancer patients. In the National Cancer Institute’s (NCI) Cancer Trends Progress Report 2015–2019, smoking rates were higher in cancer patients than in the general United States (US) population, but the trend was downwards with an annual percentage change of 1.56% (2015–2018) followed by a small rise in 2019 [25]. In our study, current smoking rates were lower among cancer patients (overall) than the concurrent rate in the general population (2015–2019) of Ireland, but the upward trend was in contrast to the national decline in smoking rates [23,26].

Based on the 2017 US National Health Interview Survey data (*n* = 26,742), Gritz et al. (2020) reported that among cancer survivors the rates of current smoking was 13.1% versus 26.1% for past smoking (smoked at least 100 cigarettes in their life but were not smoking at the time of the survey) [27] which is slightly higher than our findings of current and past smoking (11.76% and 21.02%, respectively, in 2017). Current and past smoking rates reported for individual cancers by Gritz et al. were 12.64% and 23.27% for BC and 32.24% and 30.76% for CC, respectively [27], which were much higher than the present analysis where current and past smoking was 6.11% and 10.83% for BC and 17% and 17.73% for CC, respectively. However, the rates in LC patients reported by Gritz and colleagues were much lower than in our study (10.9% vs. 27.26% and 34.43% vs. 39.09%, respectively) [27].

Continued smoking is associated with lower survival rates among cancer patients. The National Cancer Registry of Ireland reported that cancer patients who were current smokers had significantly increased mortality compared to never smokers (HR = 1.36, 95% CI: 1.21–1.52) [28]. Given the high smoking rates among cancer patients, it is critical that healthcare professionals advocate smoking cessation among all patients including those diagnosed with cancer. However, in practice it is recognised that patients do not receive sufficient encouragement from their health care providers to quit smoking [29,30]. Some of the barriers to broach the subject of smoking cessation from oncology healthcare professionals include (i) concerns around the negative impact of the therapeutic relationship, (ii) perceptions that such discussions were not appropriate [31], (ii) likely to be ineffective, [17,31] or (iii) stigmatising [31] and (v) inadequate training in smoking cessation [17,31]. Hospital smoking cessation services are imperative to smoking cessation among cancer patients, but underutilisation has also been observed. In the US, in 2012, the Joint Commission set a quality standard for hospitals and recommended that all admitted patients identified as current smokers should receive smoking cessation advice. Notwithstanding the recommendations, many hospitals failed to comply [32,33,34,35].

Motivation to stop smoking is often high immediately after diagnosis—a time that can be taken advantage of as a key ‘teachable moment’ [11]. Patient-related barriers to smoking cessation include being in a younger age group, depression, high nicotine dependence, living with someone who smokes and socio-economic difficulties [11,36], stress (related to the cancer diagnosis), a wish to maintain personal control and a sense of ‘normal’ self and being unconvinced or not accepting the connection between smoking, cancer and health [17]. Karam-Hage et al. also identified the lack of a tobacco treatment programme integrated with cancer treatment as a barrier [12]. These can be addressed by considering communication style and the need for prolonged and intensified counselling and pharmacotherapy support [11].

Early integration of general practitioners (GPs) with cancer patient care can potentiate and help to bridge the lack of onsite smoking cessation services in hospitals [37], and the advantage in smoking cessation through a GP is attributable to frequent visits by cancer patients [38]. The advice from the GP can be delivered in various formats such as very brief, brief or intensive. In a UK study of 42 clinical trials between 1972 and 2012 on smoking cessation advice including 31,000 smokers, primary care was found to be the most common site for advice; brief intervention resulted in increased quit rates [39]. Smoking cessation services in the UK encourages all GPs to identify patients who smoke and provide smoking cessation advice, following which 4-week behavioural support is also provided [40]. Smoking cessation should be viewed as an interdisciplinary intervention, interlinking primary care and hospital services [41].

The Irish National Cancer Strategy 2017–2026 stated that smoking has the biggest impact on cancer prevention. Tobacco Free Ireland, the National Tobacco Control strategy aims to make Ireland tobacco-free (prevalence of less than 5%) by 2025, and has been recognized as a crucial part of cancer prevention [42]. Daily smoking has declined to 14% among those over the age of 15 in Ireland, with a further 3% occasional smokers in 2019 [26]. The National Tobacco Control Strategy also advises on providing appropriate smoking cessation supports as part of the treatment regime and care planning for all cancer survivors [42] and recognises smoking cessation services as an important element of tobacco control [37]. The National Brief Intervention Programme (Making Every Contact Count) embraces behaviour change support as a routine part of health care practice and delivery to facilitate prevention of smoking [42]. There are a wide range of smoking cessation services provided by the Health Service Executive (HSE) in Ireland, including a QUITline phone service for cessation support, online and social media supports on Quit.ie and Facebook, primary care supports provided by GPs, pharmacists and dentists, HSE quit clinics and courses, and medication and nicotine replacement therapies [43]. The Irish Cancer Society runs ‘Daffodil Centres’ in many large hospitals in Ireland which provide onsite information, support and advice on smoking cessation as part of cancer prevention [44]. Both in-hospital smoking cessation programmes and those provided by the HSE in Ireland are not disease- or time-specific and the services available for cancer patients are the same. It is unknown how routinely or consistently smoking cessation interventions are being offered to patients with cancer. When the service is offered, rates of uptake and attendance are unclear, but anecdotally it is known that fewer cancer patients are referred.

To our knowledge, in the last ten years this is the first study from Ireland and Europe to evaluate the prevalence of smoking among cancer patients. This study has been conducted using data from the HIPE system, a computer-based information system that captures data on discharges from 62 acute public hospitals in Ireland. Among eight national health information systems, it has the largest database, contributing to the strength of the study [45]. This is an important but under-researched topic in Ireland. The data in this study will provide the basis for planning of smoking cessation services for cancer patients; the exact nature of such services will depend on the final collated results from our feasibility study.

However, there are some limitations with the use of the HIPE system. The collection of smoking-related codes is very much dependent on the doctors and nursing staff recording this information in the notes, which may not be consistent across hospitals; therefore, it is recognised that smoking documentation may be an underestimate [22]. As there is no code for ‘never smoked’ in the WHO ICD classification used for HIPE, it is difficult to ascertain the degree of underestimation of tobacco use [22]. This problem has been highlighted in other reviews also [46]. It was observed in the NCI’s clinical cancer trials that only 21.9% of clinicians assessed current smoking and 2.6% secondhand smoke exposure at enrolment and only 4.5% assessed tobacco use during follow up and 0.6% assessed secondhand smoke exposure during follow up [46]. Another limitation is that HIPE data represents the number of discharges and there is no unique identification number for patients. As patients may be admitted more than once, there may be some duplicate data [45]. However, the HIPE data are recorded for each patient admitted to public hospitals in Ireland and coded using standard international classification systems. Despite the limitation of duplicate episodes of care, as the number of discharges in each cancer group were comparable during the 5-year study period (2014–2018), this limitation should be stable over the study period and trend analysis was meaningful.

## 5. Conclusions

In conclusion, smoking trends in cancer patients show a worrying upward trend. Cancer patients being routinely asked about smoking by their healthcare professionals and its documentation is crucial to smoking cessation. Robust documentation of smoking status will also support careful evaluation of smoking among cancer patients in future studies. Referral of cancer patients who smoke to hospital-based or community smoking cessation services is pivotal to increase their chances of quitting and reduce smoking relapses. It is important that there is an accurate way to capture smoking rates in cancer patients to facilitate a national review; routine documentation of smoking status in medical and nursing charts, and improved capture in the HIPE database is imperative.

## Figures and Tables

**Table 1:** Total number of discharges with a diagnosis of cancer (all combined and individual cancer) reported by HIPE during 2014–2018.

Cancer Type	Number of Discharges Reported				
	2014	2015	2016	2017	2018
All cancers	27009	25606	26167	26527	27276
	*n* (%)	*n* (%)	*n* (%)	*n* (%)	*n* (%)
Breast	2369 (8.8)	2325 (9.1)	2250 (8.6)	2307 (8.7)	2469 (9.0)
Lung	835 (3.1)	871 (3.4)	1003 (3.8)	988 (3.7)	995 (3.6)
Cervical	426 (1.6)	402 (1.6)	320 (1.2)	389 (1.5)	387 (1.4)
Head and Neck	254 (1.0)	257 (1.0)	258 (1.0)	271 (1.0)	234 (1.0)

**Table 2:** Five-year (2014–2018) trend of current smoking among all cancer (combined), breast, lung, cervical and head and neck cancer patients overall and age- and gender-specific in Ireland.

Variables	Cancer Type	Categories	Year				
			2014	2015	2016	2017	2018
Overall current smoking rates (%)	All cancer		10.53	11.24	11.20	11.76	11.44
	Breast cancer (female)		5.90	6.62	6.44	6.11	5.71
	Lung cancer		24.74	26.53	24.02	27.26	24.11
	Cervical cancer		11.00	14.67	12.80	17.00	19.80
	Head and Neck cancer		3.14	8.56	12.79	10.33	7.69
Gender-specific rates (%)	All cancers	Male	12.08	12.45	12.43	13.07	13.00
		Female	8.81	9.92	9.83	10.31	9.72
	Breast cancer	Female	5.90	6.62	6.44	6.11	5.71
	Lung cancer	Male	26.25	27.00	22.56	27.05	24.72
		Female	22.78	25.95	25.68	27.51	23.33
	Cervical cancer	Female	11.00	14.67	12.80	17.00	19.80
	Head and Neck cancer	Male	~	9.00	16.55	8.10	~
		Female	~	8.00	7.96	13.00	~
Age-specific rates (%)	All cancers	Less than 40 years	10.60	11.08	11.77	10.37	9.97
		40–49 years	11.93	12.74	12.03	13.51	13.60
		50–59 years	14.00	14.52	15.25	15.91	15.53
		60–69 years	12.33	13.62	13.39	13.83	13.20
		70 years and over	7.36	7.58	7.53	8.29	8.08
	Breast cancer	Less than 40 years	10.50	6.95	6.48	4.39	6.62
		40–49 years	5.80	5.58	5.21	5.31	4.85
		50–59 years	6.34	8.56	8.63	7.54	7.27
		60–69 years	7.11	8.08	8.16	8.52	7.22
		70 years and over	3.08	4.05	4.09	4.02	3.34
	Lung cancer	Less than 40 years	~	~	20.68	~	~
		40–49 years	28.44	32.00	32.89	38.00	21.90
		50–59 years	35.82	35.40	32.50	34.92	34.91
		60–69 years	26.02	31.49	27.10	28.52	27.40
		70 years and over	18.08	18.21	17.53	22.27	18.21
	Cervical cancer	Less than 40 years	7.82	14.40	~	6.18	23.57
		40–59 years	12.16	19.66	14.58	21.54	25.97
		60 years and over	12.29	6.60	~	18.91	7.27
	Head and Neck cancer	Less than 40 years	~	16.36	21.73	~	~
		40–59 years	~	8.90	11.53	~	10.00
		60 years and over	~	5.18	10.44	11.47	7.20

~ denotes five or fewer cases reported to HIPE or cases that have been suppressed for reason of confidentiality and not disclosed.

**Table 3:** Five-year (2014–2018) trend of past smoking among all cancers (combined), breast, lung, cervical and head and neck cancer patients overall and by age and gender in Ireland.

Variables	Cancer Type	Categories	Year				
			2014	2015	2016	2017	2018
Overall past-smoking rates (%)	All cancer		15.22	17.46	19.70	21.02	20.75
	Breast cancer (female)		7.26	9.11	11.02	10.83	12.83
	Lung cancer		30.34	34.34	37.59	39.09	38.09
	Cervical cancer		11.26	8.95	14.06	17.73	13.17
	Head and Neck cancer		8.26	15.95	18.21	25.83	16.23
Gender-specific rates (%)	All cancers	Male	18.90	21.94	24	25.9	24.79
		Female	11.13	12.61	15	15.6	16.27
	Breast cancer	Female	7.26	9.11	11.02	10.83	12.83
	Lung cancer	Male	31.33	36.00	40.40	43.50	40.87
		Female	29.04	32.20	34.40	33.74	34.52
	Cervical cancer	Female	11.26	8.95	14.06	17.73	13.17
	Head and Neck cancer	Male	~	14.50	22.06	28.30	10.86
		Female	~	17.46	13.27	22.76	23.95
Age-specific rates (%)	All cancers	Less than 40 years	7.60	7.56	9.86	10.52	9.59
		40–49 years	11.04	10.96	14.39	14.30	14.36
		50–59 years	12.97	16.08	17.56	17.27	17.75
		60–69 years	16.87	19.15	21.50	23.68	23.22
		70 years and over	17.51	20.52	22.66	24.50	23.91
	Breast cancer	Less than 40 years	4.90	5.34	8.10	4.94	5.42
		40–49 years	8.00	6.04	11.96	11.24	12.86
		50–59 years	7.01	10.13	10.13	9.12	10.53
		60–69 years	8.89	12.13	11.47	12.79	16.26
		70 years and over	6.17	8.78	11.58	12.39	14.45
	Lung cancer	Less than 40 years	~	~	20.68	~	~
		40–49 years	~	~	23.68	28.00	35.23
		50–59 years	21.76	29.19	33.00	22.68	21.22
		60–69 years	30.87	32.92	36.70	38.25	37.84
		70 years and over	34.28	39.08	41.06	43.69	41.74
	Cervical cancer	Less than 40 years	13.04	5.93	11.81	21.64	10.56
		40–59 years	12.16	11.23	13.88	13.25	13.63
		60 years and over	8.19	8.49	18.18	21.62	15.45
	Head and Neck cancer	Less than 40 years	~	~	~	20.00	~
		40–59 years	~	~	17.94	24.46	20.00
		60 years and over	9.00	25.18	24.62	29.50	~

~ denotes five or fewer cases reported to HIPE or cases that have been suppressed for reason of confidentiality and not disclosed.

## Data Availability

Data were obtained from the HPO, Ireland https://www.hiqa.ie/areas-we-work/health-information/data-collections/hospital-patient-enquiry-hipe (accessed on 10 February 2022) and are available from the authors with the permission of the HPO.
